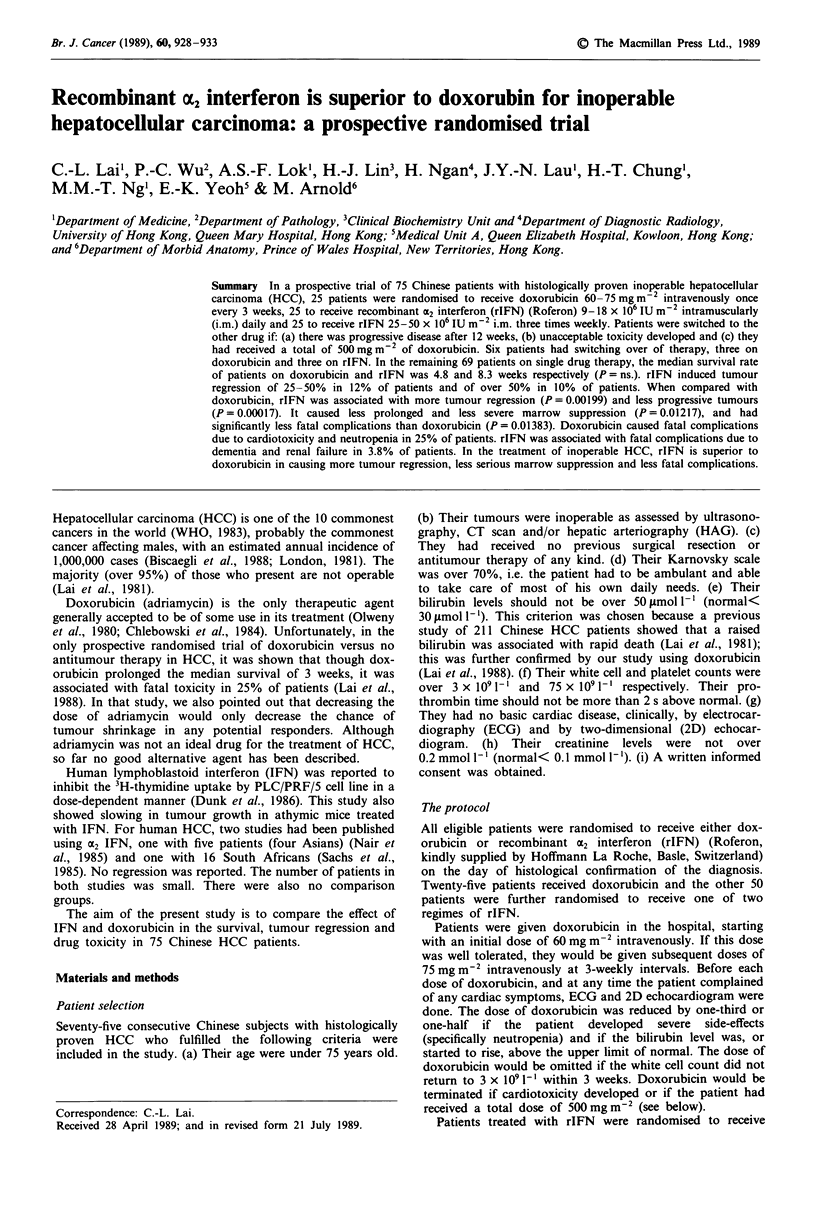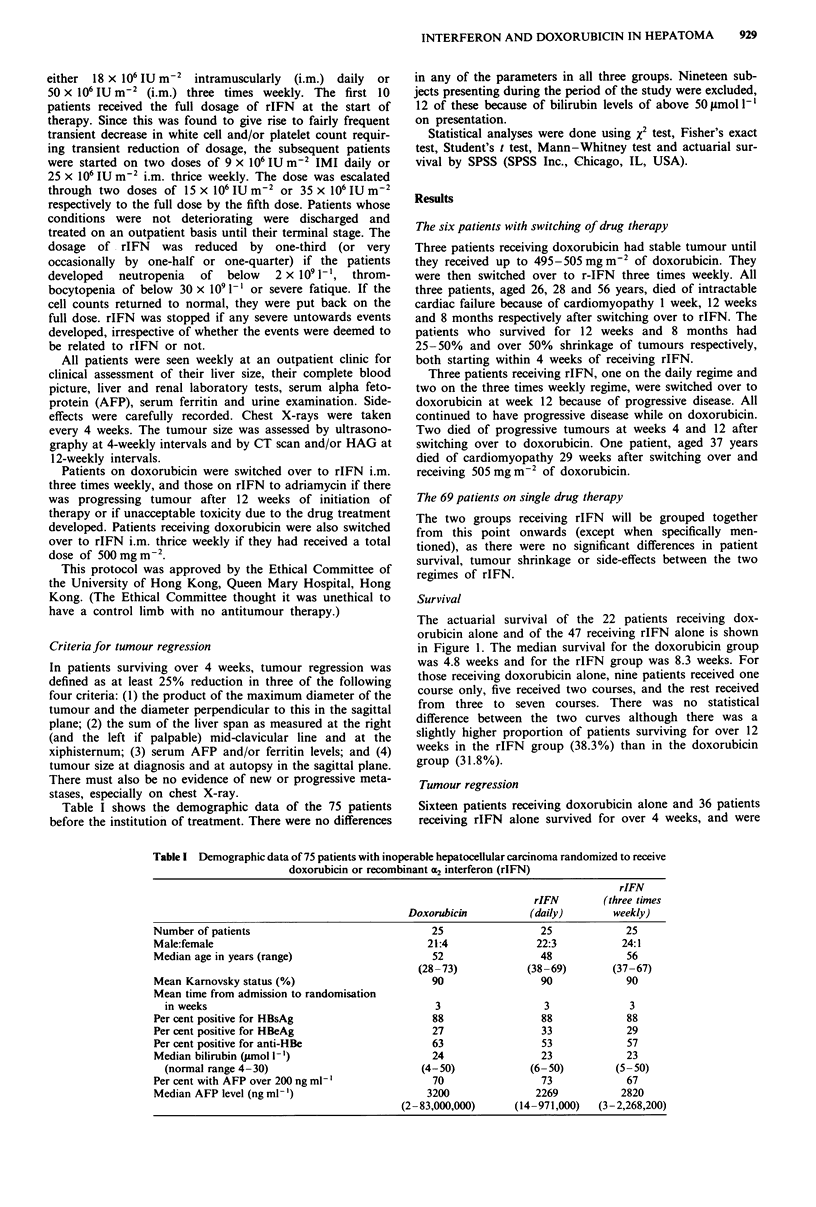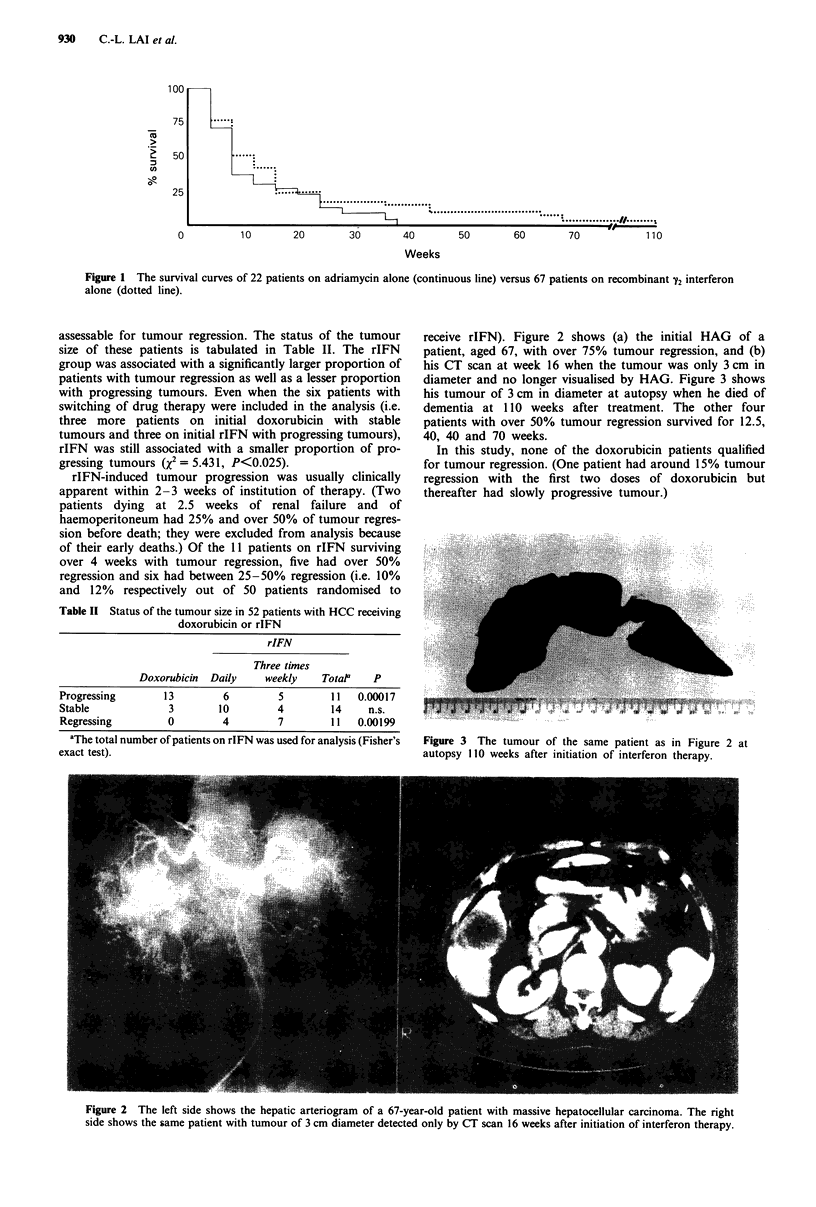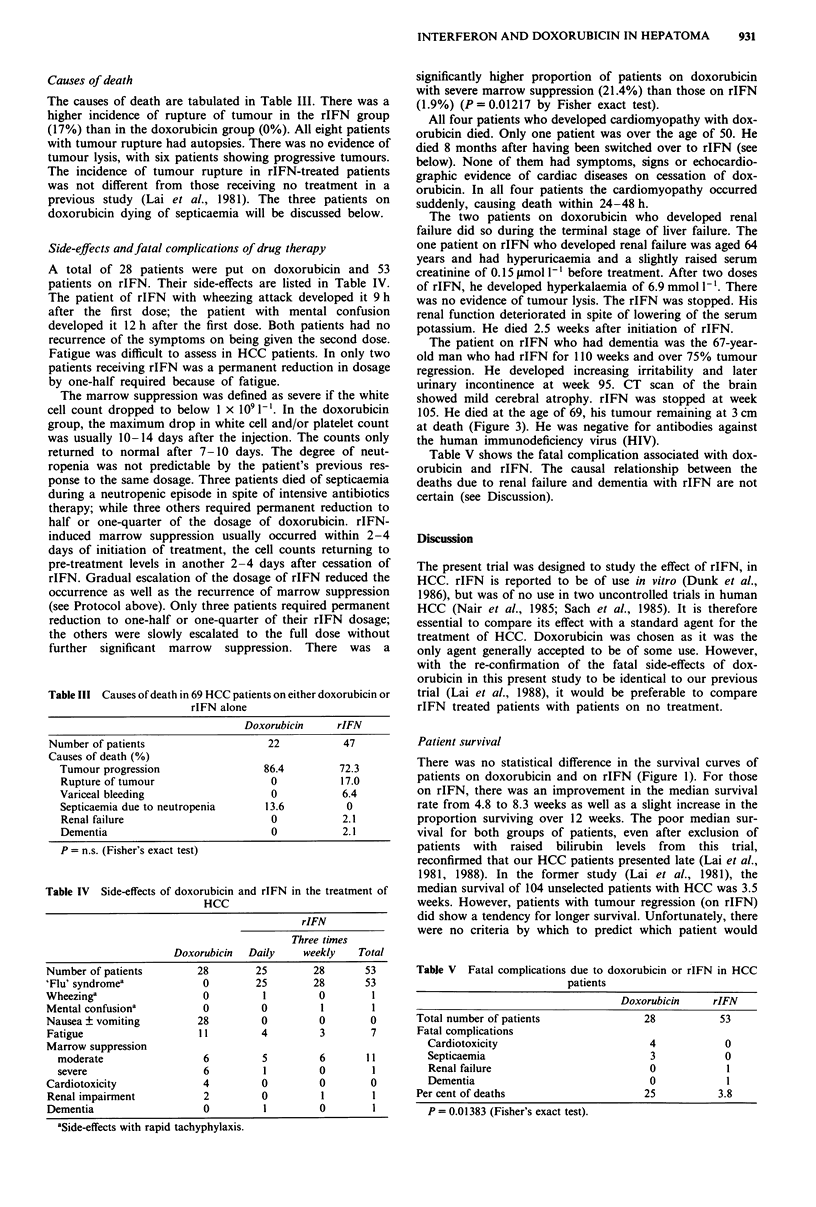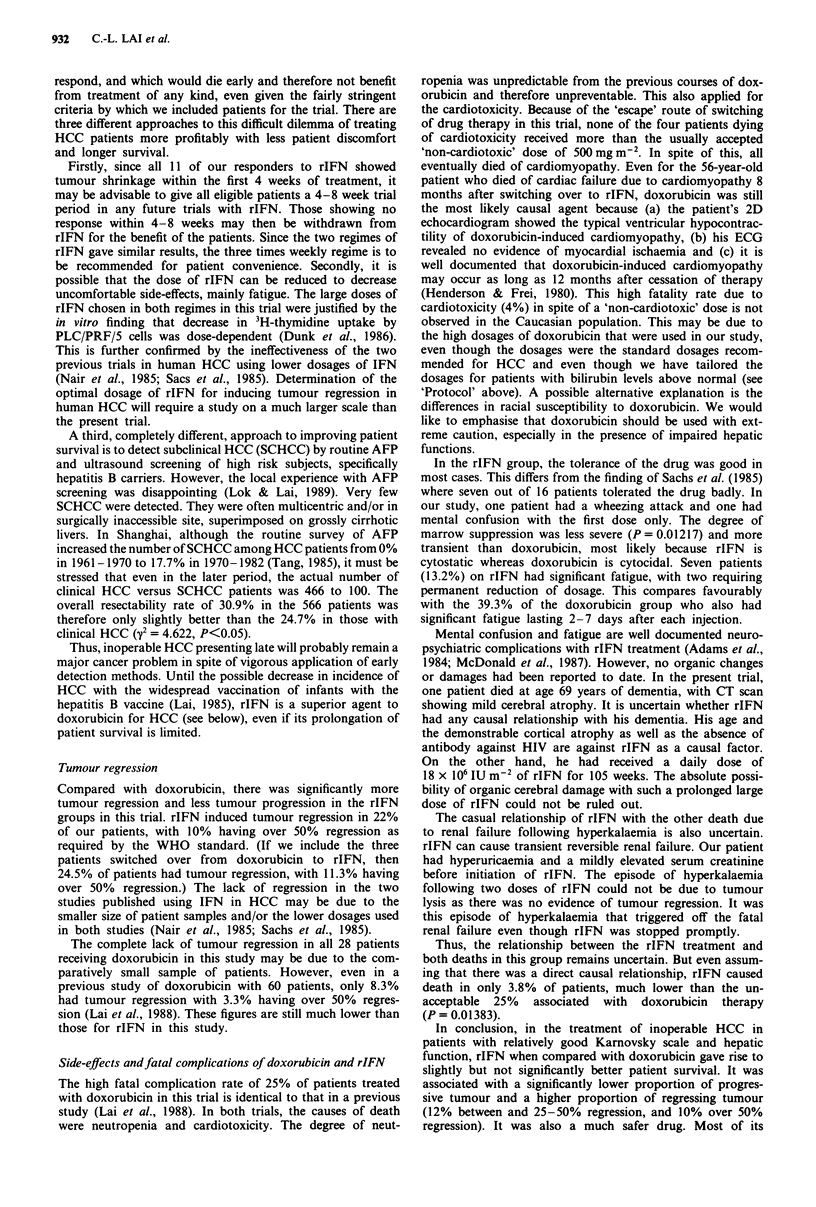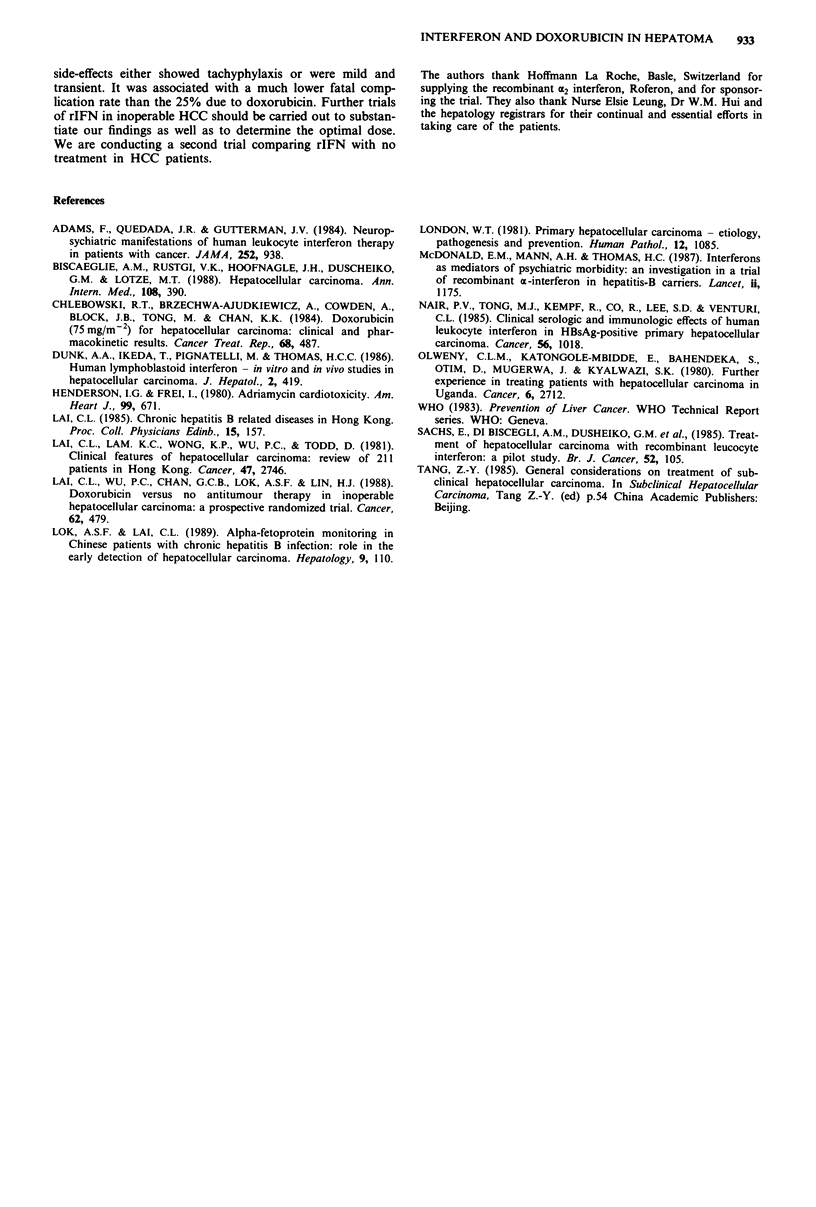# Recombinant alpha 2 interferon is superior to doxorubicin for inoperable hepatocellular carcinoma: a prospective randomised trial.

**DOI:** 10.1038/bjc.1989.392

**Published:** 1989-12

**Authors:** C. L. Lai, P. C. Wu, A. S. Lok, H. J. Lin, H. Ngan, J. Y. Lau, H. T. Chung, M. M. Ng, E. K. Yeoh, M. Arnold

**Affiliations:** Department of Medicine, University of Hong Kong, Queen Mary Hospital.

## Abstract

**Images:**


*Br. J. Cancer* (1989), **60**, 928–933 © The Macmillan Press Ltd., 1989

### Recombinant $\alpha_2$ interferon is superior to doxorubin for inoperable hepatocellular carcinoma: a prospective randomised trial

C.-L. Lai[1], P.-C. Wu[2], A.S.-F. Lok[1], H.-J. Lin[3], H. Ngan[4], J.Y.-N. Lau[1], H.-T. Chung[1], M.M.-T. Ng[1], E.-K. Yeoh[5] & M. Arnold[6]

[1]*Department of Medicine,* [2]*Department of Pathology,* [3]*Clinical Biochemistry Unit and* [4]*Department of Diagnostic Radiology, University of Hong Kong, Queen Mary Hospital, Hong Kong;* [5]*Medical Unit A, Queen Elizabeth Hospital, Kowloon, Hong Kong; and* [6]*Department of Morbid Anatomy, Prince of Wales Hospital, New Territories, Hong Kong.*

**Summary** In a prospective trial of 75 Chinese patients with histologically proven inoperable hepatocellular carcinoma (HCC), 25 patients were randomised to receive doxorubicin 60–75 mg m$^{-2}$ intravenously once every 3 weeks, 25 to receive recombinant $\alpha_2$ interferon (rIFN) (Roferon) 9–18 × 10$^6$ IU m$^{-2}$ intramuscularly (i.m.) daily and 25 to receive rIFN 25–50 × 10$^6$ IU m$^{-2}$ i.m. three times weekly. Patients were switched to the other drug if: (a) there was progressive disease after 12 weeks, (b) unacceptable toxicity developed and (c) they had received a total of 500 mg m$^{-2}$ of doxorubicin. Six patients had switching over of therapy, three on doxorubicin and three on rIFN. In the remaining 69 patients on single drug therapy, the median survival rate of patients on doxorubicin and rIFN was 4.8 and 8.3 weeks respectively ($P$ = ns.). rIFN induced tumour regression of 25–50% in 12% of patients and of over 50% in 10% of patients. When compared with doxorubicin, rIFN was associated with more tumour regression ($P = 0.00199$) and less progressive tumours ($P = 0.00017$). It caused less prolonged and less severe marrow suppression ($P = 0.01217$), and had significantly less fatal complications than doxorubicin ($P = 0.01383$). Doxorubicin caused fatal complications due to cardiotoxicity and neutropenia in 25% of patients. rIFN was associated with fatal complications due to dementia and renal failure in 3.8% of patients. In the treatment of inoperable HCC, rIFN is superior to doxorubicin in causing more tumour regression, less serious marrow suppression and less fatal complications.

Hepatocellular carcinoma (HCC) is one of the 10 commonest cancers in the world (WHO, 1983), probably the commonest cancer affecting males, with an estimated annual incidence of 1,000,000 cases (Biscaegli *et al.*, 1988; London, 1981). The majority (over 95%) of those who present are not operable (Lai *et al.*, 1981).

Doxorubicin (adriamycin) is the only therapeutic agent generally accepted to be of some use in its treatment (Olweny *et al.*, 1980; Chlebowski *et al.*, 1984). Unfortunately, in the only prospective randomised trial of doxorubicin versus no antitumour therapy in HCC, it was shown that though doxorubicin prolonged the median survival of 3 weeks, it was associated with fatal toxicity in 25% of patients (Lai *et al.*, 1988). In that study, we also pointed out that decreasing the dose of adriamycin would only decrease the chance of tumour shrinkage in any potential responders. Although adriamycin was not an ideal drug for the treatment of HCC, so far no good alternative agent has been described.

Human lymphoblastoid interferon (IFN) was reported to inhibit the $^3$H-thymidine uptake by PLC/PRF/5 cell line in a dose-dependent manner (Dunk *et al.*, 1986). This study also showed slowing in tumour growth in athymic mice treated with IFN. For human HCC, two studies had been published using $\alpha_2$ IFN, one with five patients (four Asians) (Nair *et al.*, 1985) and one with 16 South Africans (Sachs *et al.*, 1985). No regression was reported. The number of patients in both studies was small. There were also no comparison groups.

The aim of the present study is to compare the effect of IFN and doxorubicin in the survival, tumour regression and drug toxicity in 75 Chinese HCC patients.

### Materials and methods

### *Patient selection*

Seventy-five consecutive Chinese subjects with histologically proven HCC who fulfilled the following criteria were included in the study. (a) Their age were under 75 years old. (b) Their tumours were inoperable as assessed by ultrasonography, CT scan and/or hepatic arteriography (HAG). (c) They had received no previous surgical resection or antitumour therapy of any kind. (d) Their Karnovsky scale was over 70%, i.e. the patient had to be ambulant and able to take care of most of his own daily needs. (e) Their bilirubin levels should not be over 50 µmol l$^{-1}$ (normal< 30 µmol l$^{-1}$). This criterion was chosen because a previous study of 211 Chinese HCC patients showed that a raised bilirubin was associated with rapid death (Lai *et al.*, 1981); this was further confirmed by our study using doxorubicin (Lai *et al.*, 1988). (f) Their white cell and platelet counts were over 3 × 10$^9$ l$^{-1}$ and 75 × 10$^9$ l$^{-1}$ respectively. Their prothrombin time should not be more than 2 s above normal. (g) They had no basic cardiac disease, clinically, by electrocardiography (ECG) and by two-dimensional (2D) echocardiogram. (h) Their creatinine levels were not over 0.2 mmol l$^{-1}$ (normal< 0.1 mmol l$^{-1}$). (i) A written informed consent was obtained.

### *The protocol*

All eligible patients were randomised to receive either doxorubicin or recombinant $\alpha_2$ interferon (rIFN) (Roferon, kindly supplied by Hoffmann La Roche, Basle, Switzerland) on the day of histological confirmation of the diagnosis. Twenty-five patients received doxorubicin and the other 50 patients were further randomised to receive one of two regimes of rIFN.

Patients were given doxorubicin in the hospital, starting with an initial dose of 60 mg m$^{-2}$ intravenously. If this dose was well tolerated, they would be given subsequent doses of 75 mg m$^{-2}$ intravenously at 3-weekly intervals. Before each dose of doxorubicin, and at any time the patient complained of any cardiac symptoms, ECG and 2D echocardiogram were done. The dose of doxorubicin was reduced by one-third or one-half if the patient developed severe side-effects (specifically neutropenia) and if the bilirubin level was, or started to rise, above the upper limit of normal. The dose of doxorubicin would be omitted if the white cell count did not return to 3 × 10$^9$ l$^{-1}$ within 3 weeks. Doxorubicin would be terminated if cardiotoxicity developed or if the patient had received a total dose of 500 mg m$^{-2}$ (see below).

Patients treated with rIFN were randomised to receive

Correspondence: C.-L. Lai.
Received 28 April 1989; and in revised form 21 July 1989.

either $18 \times 10^6$ IU m$^{-2}$ intramuscularly (i.m.) daily or $50 \times 10^6$ IU m$^{-2}$ (i.m.) three times weekly. The first 10 patients received the full dosage of rIFN at the start of therapy. Since this was found to give rise to fairly frequent transient decrease in white cell and/or platelet count requiring transient reduction of dosage, the subsequent patients were started on two doses of $9 \times 10^6$ IU m$^{-2}$ IMI daily or $25 \times 10^6$ IU m$^{-2}$ i.m. thrice weekly. The dose was escalated through two doses of $15 \times 10^6$ IU m$^{-2}$ or $35 \times 10^6$ IU m$^{-2}$ respectively to the full dose by the fifth dose. Patients whose conditions were not deteriorating were discharged and treated on an outpatient basis until their terminal stage. The dosage of rIFN was reduced by one-third (or very occasionally by one-half or one-quarter) if the patients developed neutropenia of below $2 \times 10^9$ l$^{-1}$, thrombocytopenia of below $30 \times 10^9$ l$^{-1}$ or severe fatique. If the cell counts returned to normal, they were put back on the full dose. rIFN was stopped if any severe untowards events developed, irrespective of whether the events were deemed to be related to rIFN or not.

All patients were seen weekly at an outpatient clinic for clinical assessment of their liver size, their complete blood picture, liver and renal laboratory tests, serum alpha fetoprotein (AFP), serum ferritin and urine examination. Side-effects were carefully recorded. Chest X-rays were taken every 4 weeks. The tumour size was assessed by ultrasonography at 4-weekly intervals and by CT scan and/or HAG at 12-weekly intervals.

Patients on doxorubicin were switched over to rIFN i.m. three times weekly, and those on rIFN to adriamycin if there was progressing tumour after 12 weeks of initiation of therapy or if unacceptable toxicity due to the drug treatment developed. Patients receiving doxorubicin were also switched over to rIFN i.m. thrice weekly if they had received a total dose of 500 mg m$^{-2}$.

This protocol was approved by the Ethical Committee of the University of Hong Kong, Queen Mary Hospital, Hong Kong. (The Ethical Committee thought it was unethical to have a control limb with no antitumour therapy.)

### *Criteria for tumour regression*

In patients surviving over 4 weeks, tumour regression was defined as at least 25% reduction in three of the following four criteria: (1) the product of the maximum diameter of the tumour and the diameter perpendicular to this in the sagittal plane; (2) the sum of the liver span as measured at the right (and the left if palpable) mid-clavicular line and at the xiphisternum; (3) serum AFP and/or ferritin levels; and (4) tumour size at diagnosis and at autopsy in the sagittal plane. There must also be no evidence of new or progressive metastases, especially on chest X-ray.

Table I shows the demographic data of the 75 patients before the institution of treatment. There were no differences in any of the parameters in all three groups. Nineteen subjects presenting during the period of the study were excluded, 12 of these because of bilirubin levels of above 50 $\mu$mol l$^{-1}$ on presentation.

Statistical analyses were done using $\chi^2$ test, Fisher's exact test, Student's $t$ test, Mann-Whitney test and actuarial survival by SPSS (SPSS Inc., Chicago, IL, USA).

### Results

### *The six patients with switching of drug therapy*

Three patients receiving doxorubicin had stable tumour until they received up to 495–505 mg m$^{-2}$ of doxorubicin. They were then switched over to r-IFN three times weekly. All three patients, aged 26, 28 and 56 years, died of intractable cardiac failure because of cardiomyopathy 1 week, 12 weeks and 8 months respectively after switching over to rIFN. The patients who survived for 12 weeks and 8 months had 25–50% and over 50% shrinkage of tumours respectively, both starting within 4 weeks of receiving rIFN.

Three patients receiving rIFN, one on the daily regime and two on the three times weekly regime, were switched over to doxorubicin at week 12 because of progressive disease. All continued to have progressive disease while on doxorubicin. Two died of progressive tumours at weeks 4 and 12 after switching over to doxorubicin. One patient, aged 37 years died of cardiomyopathy 29 weeks after switching over and receiving 505 mg m$^{-2}$ of doxorubicin.

### *The 69 patients on single drug therapy*

The two groups receiving rIFN will be grouped together from this point onwards (except when specifically mentioned), as there were no significant differences in patient survival, tumour shrinkage or side-effects between the two regimes of rIFN.

### *Survival*

The actuarial survival of the 22 patients receiving doxorubicin alone and of the 47 receiving rIFN alone is shown in Figure 1. The median survival for the doxorubicin group was 4.8 weeks and for the rIFN group was 8.3 weeks. For those receiving doxorubicin alone, nine patients received one course only, five received two courses, and the rest received from three to seven courses. There was no statistical difference between the two curves although there was a slightly higher proportion of patients surviving for over 12 weeks in the rIFN group (38.3%) than in the doxorubicin group (31.8%).

### *Tumour regression*

Sixteen patients receiving doxorubicin alone and 36 patients receiving rIFN alone survived for over 4 weeks, and were

**Table I** Demographic data of 75 patients with inoperable hepatocellular carcinoma randomized to receive doxorubicin or recombinant $\alpha_2$ interferon (rIFN)

| | *Doxorubicin* | *rIFN (daily)* | *rIFN (three times weekly)* |
|---|---|---|---|
| Number of patients | 25 | 25 | 25 |
| Male:female | 21:4 | 22:3 | 24:1 |
| Median age in years (range) | 52 (28–73) | 48 (38–69) | 56 (37–67) |
| Mean Karnovsky status (%) | 90 | 90 | 90 |
| Mean time from admission to randomisation in weeks | 3 | 3 | 3 |
| Per cent positive for HBsAg | 88 | 88 | 88 |
| Per cent positive for HBeAg | 27 | 33 | 29 |
| Per cent positive for anti-HBe | 63 | 53 | 57 |
| Median bilirubin ($\mu$mol l$^{-1}$) (normal range 4–30) | 24 (4–50) | 23 (6–50) | 23 (5–50) |
| Per cent with AFP over 200 ng ml$^{-1}$ | 70 | 73 | 67 |
| Median AFP level (ng ml$^{-1}$) | 3200 (2–83,000,000) | 2269 (14–971,000) | 2820 (3–2,268,200) |

**Figure 1** The survival curves of 22 patients on adriamycin alone (continuous line) versus 67 patients on recombinant $\gamma_2$ interferon alone (dotted line).

assessable for tumour regression. The status of the tumour size of these patients is tabulated in Table II. The rIFN group was associated with a significantly larger proportion of patients with tumour regression as well as a lesser proportion with progressing tumours. Even when the six patients with switching of drug therapy were included in the analysis (i.e. three more patients on initial doxorubicin with stable tumours and three on initial rIFN with progressing tumours), rIFN was still associated with a smaller proportion of progressing tumours ($\chi^2 = 5.431$, $P<0.025$).

rIFN-induced tumour progression was usually clinically apparent within 2–3 weeks of institution of therapy. (Two patients dying at 2.5 weeks of renal failure and of haemoperitoneum had 25% and over 50% of tumour regression before death; they were excluded from analysis because of their early deaths.) Of the 11 patients on rIFN surviving over 4 weeks with tumour regression, five had over 50% regression and six had between 25–50% regression (i.e. 10% and 12% respectively out of 50 patients randomised to

**Table II** Status of the tumour size in 52 patients with HCC receiving doxorubicin or rIFN

| | | *rIFN* | | | |
|---|---|---|---|---|---|
| | *Doxorubicin* | *Daily* | *Three times weekly* | *Total*[a] | *P* |
| Progressing | 13 | 6 | 5 | 11 | 0.00017 |
| Stable | 3 | 10 | 4 | 14 | n.s. |
| Regressing | 0 | 4 | 7 | 11 | 0.00199 |

[a]The total number of patients on rIFN was used for analysis (Fisher's exact test).

receive rIFN). Figure 2 shows (a) the initial HAG of a patient, aged 67, with over 75% tumour regression, and (b) his CT scan at week 16 when the tumour was only 3 cm in diameter and no longer visualised by HAG. Figure 3 shows his tumour of 3 cm in diameter at autopsy when he died of dementia at 110 weeks after treatment. The other four patients with over 50% tumour regression survived for 12.5, 40, 40 and 70 weeks.

In this study, none of the doxorubicin patients qualified for tumour regression. (One patient had around 15% tumour regression with the first two doses of doxorubicin but thereafter had slowly progressive tumour.)

**Figure 3** The tumour of the same patient as in Figure 2 at autopsy 110 weeks after initiation of interferon therapy.

**Figure 2** The left side shows the hepatic arteriogram of a 67-year-old patient with massive hepatocellular carcinoma. The right side shows the same patient with tumour of 3 cm diameter detected only by CT scan 16 weeks after initiation of interferon therapy.

*Causes of death*

The causes of death are tabulated in Table III. There was a higher incidence of rupture of tumour in the rIFN group (17%) than in the doxorubicin group (0%). All eight patients with tumour rupture had autopsies. There was no evidence of tumour lysis, with six patients showing progressive tumours. The incidence of tumour rupture in rIFN-treated patients was not different from those receiving no treatment in a previous study (Lai *et al.*, 1981). The three patients on doxorubicin dying of septicaemia will be discussed below.

*Side-effects and fatal complications of drug therapy*

A total of 28 patients were put on doxorubicin and 53 patients on rIFN. Their side-effects are listed in Table IV. The patient of rIFN with wheezing attack developed it 9 h after the first dose; the patient with mental confusion developed it 12 h after the first dose. Both patients had no recurrence of the symptoms on being given the second dose. Fatigue was difficult to assess in HCC patients. In only two patients receiving rIFN was a permanent reduction in dosage by one-half required because of fatigue.

The marrow suppression was defined as severe if the white cell count dropped to below $1 \times 10^9 l^{-1}$. In the doxorubicin group, the maximum drop in white cell and/or platelet count was usually 10–14 days after the injection. The counts only returned to normal after 7–10 days. The degree of neutropenia was not predictable by the patient's previous response to the same dosage. Three patients died of septicaemia during a neutropenic episode in spite of intensive antibiotics therapy; while three others required permanent reduction to half or one-quarter of the dosage of doxorubicin. rIFN-induced marrow suppression usually occurred within 2–4 days of initiation of treatment, the cell counts returning to pre-treatment levels in another 2–4 days after cessation of rIFN. Gradual escalation of the dosage of rIFN reduced the occurrence as well as the recurrence of marrow suppression (see Protocol above). Only three patients required permanent reduction to one-half or one-quarter of their rIFN dosage; the others were slowly escalated to the full dose without further significant marrow suppression. There was a significantly higher proportion of patients on doxorubicin with severe marrow suppression (21.4%) than those on rIFN (1.9%) ($P = 0.01217$ by Fisher exact test).

All four patients who developed cardiomyopathy with doxorubicin died. Only one patient was over the age of 50. He died 8 months after having been switched over to rIFN (see below). None of them had symptoms, signs or echocardiographic evidence of cardiac diseases on cessation of doxorubicin. In all four patients the cardiomyopathy occurred suddenly, causing death within 24–48 h.

The two patients on doxorubicin who developed renal failure did so during the terminal stage of liver failure. The one patient on rIFN who developed renal failure was aged 64 years and had hyperuricaemia and a slightly raised serum creatinine of $0.15\ \mu mol\ l^{-1}$ before treatment. After two doses of rIFN, he developed hyperkalaemia of $6.9\ mmol\ l^{-1}$. There was no evidence of tumour lysis. The rIFN was stopped. His renal function deteriorated in spite of lowering of the serum potassium. He died 2.5 weeks after initiation of rIFN.

The patient on rIFN who had dementia was the 67-year-old man who had rIFN for 110 weeks and over 75% tumour regression. He developed increasing irritability and later urinary incontinence at week 95. CT scan of the brain showed mild cerebral atrophy. rIFN was stopped at week 105. He died at the age of 69, his tumour remaining at 3 cm at death (Figure 3). He was negative for antibodies against the human immunodeficiency virus (HIV).

Table V shows the fatal complication associated with doxorubicin and rIFN. The causal relationship between the deaths due to renal failure and dementia with rIFN are not certain (see Discussion).

**Table III** Causes of death in 69 HCC patients on either doxorubicin or rIFN alone

| | *Doxorubicin* | *rIFN* |
|---|---|---|
| Number of patients | 22 | 47 |
| Causes of death (%) | | |
| Tumour progression | 86.4 | 72.3 |
| Rupture of tumour | 0 | 17.0 |
| Variceal bleeding | 0 | 6.4 |
| Septicaemia due to neutropenia | 13.6 | 0 |
| Renal failure | 0 | 2.1 |
| Dementia | 0 | 2.1 |

$P$ = n.s. (Fisher's exact test)

**Table IV** Side-effects of doxorubicin and rIFN in the treatment of HCC

| | | *rIFN* | | |
|---|---|---|---|---|
| | *Doxorubicin* | *Daily* | *Three times weekly* | *Total* |
| Number of patients | 28 | 25 | 28 | 53 |
| 'Flu' syndrome[a] | 0 | 25 | 28 | 53 |
| Wheezing[a] | 0 | 1 | 0 | 1 |
| Mental confusion[a] | 0 | 0 | 1 | 1 |
| Nausea ± vomiting | 28 | 0 | 0 | 0 |
| Fatigue | 11 | 4 | 3 | 7 |
| Marrow suppression | | | | |
| moderate | 6 | 5 | 6 | 11 |
| severe | 6 | 1 | 0 | 1 |
| Cardiotoxicity | 4 | 0 | 0 | 0 |
| Renal impairment | 2 | 0 | 1 | 1 |
| Dementia | 0 | 1 | 0 | 1 |

[a]Side-effects with rapid tachyphylaxis.

**Discussion**

The present trial was designed to study the effect of rIFN, in HCC. rIFN is reported to be of use *in vitro* (Dunk *et al.*, 1986), but was of no use in two uncontrolled trials in human HCC (Nair *et al.*, 1985; Sach *et al.*, 1985). It is therefore essential to compare its effect with a standard agent for the treatment of HCC. Doxorubicin was chosen as it was the only agent generally accepted to be of some use. However, with the re-confirmation of the fatal side-effects of doxorubicin in this present study to be identical to our previous trial (Lai *et al.*, 1988), it would be preferable to compare rIFN treated patients with patients on no treatment.

*Patient survival*

There was no statistical difference in the survival curves of patients on doxorubicin and on rIFN (Figure 1). For those on rIFN, there was an improvement in the median survival rate from 4.8 to 8.3 weeks as well as a slight increase in the proportion surviving over 12 weeks. The poor median survival for both groups of patients, even after exclusion of patients with raised bilirubin levels from this trial, reconfirmed that our HCC patients presented late (Lai *et al.*, 1981, 1988). In the former study (Lai *et al.*, 1981), the median survival of 104 unselected patients with HCC was 3.5 weeks. However, patients with tumour regression (on rIFN) did show a tendency for longer survival. Unfortunately, there were no criteria by which to predict which patient would

**Table V** Fatal complications due to doxorubicin or rIFN in HCC patients

| | *Doxorubicin* | *rIFN* |
|---|---|---|
| Total number of patients | 28 | 53 |
| Fatal complications | | |
| Cardiotoxicity | 4 | 0 |
| Septicaemia | 3 | 0 |
| Renal failure | 0 | 1 |
| Dementia | 0 | 1 |
| Per cent of deaths | 25 | 3.8 |

$P = 0.01383$ (Fisher's exact test).

respond, and which would die early and therefore not benefit from treatment of any kind, even given the fairly stringent criteria by which we included patients for the trial. There are three different approaches to this difficult dilemma of treating HCC patients more profitably with less patient discomfort and longer survival.

Firstly, since all 11 of our responders to rIFN showed tumour shrinkage within the first 4 weeks of treatment, it may be advisable to give all eligible patients a 4–8 week trial period in any future trials with rIFN. Those showing no response within 4–8 weeks may then be withdrawn from rIFN for the benefit of the patients. Since the two regimes of rIFN gave similar results, the three times weekly regime is to be recommended for patient convenience. Secondly, it is possible that the dose of rIFN can be reduced to decrease uncomfortable side-effects, mainly fatigue. The large doses of rIFN chosen in both regimes in this trial were justified by the *in vitro* finding that decrease in $^{3}$H-thymidine uptake by PLC/PRF/5 cells was dose-dependent (Dunk *et al.*, 1986). This is further confirmed by the ineffectiveness of the two previous trials in human HCC using lower dosages of IFN (Nair *et al.*, 1985; Sacs *et al.*, 1985). Determination of the optimal dosage of rIFN for inducing tumour regression in human HCC will require a study on a much larger scale than the present trial.

A third, completely different, approach to improving patient survival is to detect subclinical HCC (SCHCC) by routine AFP and ultrasound screening of high risk subjects, specifically hepatitis B carriers. However, the local experience with AFP screening was disappointing (Lok & Lai, 1989). Very few SCHCC were detected. They were often multicentric and/or in surgically inaccessible site, superimposed on grossly cirrhotic livers. In Shanghai, although the routine survey of AFP increased the number of SCHCC among HCC patients from 0% in 1961–1970 to 17.7% in 1970–1982 (Tang, 1985), it must be stressed that even in the later period, the actual number of clinical HCC versus SCHCC patients was 466 to 100. The overall resectability rate of 30.9% in the 566 patients was therefore only slightly better than the 24.7% in those with clinical HCC ($\gamma^2 = 4.622$, $P<0.05$).

Thus, inoperable HCC presenting late will probably remain a major cancer problem in spite of vigorous application of early detection methods. Until the possible decrease in incidence of HCC with the widespread vaccination of infants with the hepatitis B vaccine (Lai, 1985), rIFN is a superior agent to doxorubicin for HCC (see below), even if its prolongation of patient survival is limited.

### *Tumour regression*

Compared with doxorubicin, there was significantly more tumour regression and less tumour progression in the rIFN groups in this trial. rIFN induced tumour regression in 22% of our patients, with 10% having over 50% regression as required by the WHO standard. (If we include the three patients switched over from doxorubicin to rIFN, then 24.5% of patients had tumour regression, with 11.3% having over 50% regression.) The lack of regression in the two studies published using IFN in HCC may be due to the smaller size of patient samples and/or the lower dosages used in both studies (Nair *et al.*, 1985; Sachs *et al.*, 1985).

The complete lack of tumour regression in all 28 patients receiving doxorubicin in this study may be due to the comparatively small sample of patients. However, even in a previous study of doxorubicin with 60 patients, only 8.3% had tumour regression with 3.3% having over 50% regression (Lai *et al.*, 1988). These figures are still much lower than those for rIFN in this study.

### *Side-effects and fatal complications of doxorubicin and rIFN*

The high fatal complication rate of 25% of patients treated with doxorubicin in this trial is identical to that in a previous study (Lai *et al.*, 1988). In both trials, the causes of death were neutropenia and cardiotoxicity. The degree of neutropenia was unpredictable from the previous courses of doxorubicin and therefore unpreventable. This also applied for the cardiotoxicity. Because of the 'escape' route of switching of drug therapy in this trial, none of the four patients dying of cardiotoxicity received more than the usually accepted 'non-cardiotoxic' dose of 500 mg m$^{-2}$. In spite of this, all eventually died of cardiomyopathy. Even for the 56-year-old patient who died of cardiac failure due to cardiomyopathy 8 months after switching over to rIFN, doxorubicin was still the most likely causal agent because (a) the patient's 2D echocardiogram showed the typical ventricular hypocontractility of doxorubicin-induced cardiomyopathy, (b) his ECG revealed no evidence of myocardial ischaemia and (c) it is well documented that doxorubicin-induced cardiomyopathy may occur as long as 12 months after cessation of therapy (Henderson & Frei, 1980). This high fatality rate due to cardiotoxicity (4%) in spite of a 'non-cardiotoxic' dose is not observed in the Caucasian population. This may be due to the high dosages of doxorubicin that were used in our study, even though the dosages were the standard dosages recommended for HCC and even though we have tailored the dosages for patients with bilirubin levels above normal (see 'Protocol' above). A possible alternative explanation is the differences in racial susceptibility to doxorubicin. We would like to emphasise that doxorubicin should be used with extreme caution, especially in the presence of impaired hepatic functions.

In the rIFN group, the tolerance of the drug was good in most cases. This differs from the finding of Sachs *et al.* (1985) where seven out of 16 patients tolerated the drug badly. In our study, one patient had a wheezing attack and one had mental confusion with the first dose only. The degree of marrow suppression was less severe ($P = 0.01217$) and more transient than doxorubicin, most likely because rIFN is cytostatic whereas doxorubicin is cytocidal. Seven patients (13.2%) on rIFN had significant fatigue, with two requiring permanent reduction of dosage. This compares favourably with the 39.3% of the doxorubicin group who also had significant fatigue lasting 2–7 days after each injection.

Mental confusion and fatigue are well documented neuropsychiatric complications with rIFN treatment (Adams *et al.*, 1984; McDonald *et al.*, 1987). However, no organic changes or damages had been reported to date. In the present trial, one patient died at age 69 years of dementia, with CT scan showing mild cerebral atrophy. It is uncertain whether rIFN had any causal relationship with his dementia. His age and the demonstrable cortical atrophy as well as the absence of antibody against HIV are against rIFN as a causal factor. On the other hand, he had received a daily dose of $18 \times 10^{6}$ IU m$^{-2}$ of rIFN for 105 weeks. The absolute possibility of organic cerebral damage with such a prolonged large dose of rIFN could not be ruled out.

The casual relationship of rIFN with the other death due to renal failure following hyperkalaemia is also uncertain. rIFN can cause transient reversible renal failure. Our patient had hyperuricaemia and a mildly elevated serum creatinine before initiation of rIFN. The episode of hyperkalaemia following two doses of rIFN could not be due to tumour lysis as there was no evidence of tumour regression. It was this episode of hyperkalaemia that triggered off the fatal renal failure even though rIFN was stopped promptly.

Thus, the relationship between the rIFN treatment and both deaths in this group remains uncertain. But even assuming that there was a direct causal relationship, rIFN caused death in only 3.8% of patients, much lower than the unacceptable 25% associated with doxorubicin therapy ($P = 0.01383$).

In conclusion, in the treatment of inoperable HCC in patients with relatively good Karnovsky scale and hepatic function, rIFN when compared with doxorubicin gave rise to slightly but not significantly better patient survival. It was associated with a significantly lower proportion of progressive tumour and a higher proportion of regressing tumour (12% between and 25–50% regression, and 10% over 50% regression). It was also a much safer drug. Most of its side-effects either showed tachyphylaxis or were mild and transient. It was associated with a much lower fatal complication rate than the 25% due to doxorubicin. Further trials of rIFN in inoperable HCC should be carried out to substantiate our findings as well as to determine the optimal dose. We are conducting a second trial comparing rIFN with no treatment in HCC patients.

The authors thank Hoffmann La Roche, Basle, Switzerland for supplying the recombinant $\alpha_2$ interferon, Roferon, and for sponsoring the trial. They also thank Nurse Elsie Leung, Dr W.M. Hui and the hepatology registrars for their continual and essential efforts in taking care of the patients.

### References

ADAMS, F., QUEDADA, J.R. & GUTTERMAN, J.V. (1984). Neuropsychiatric manifestations of human leukocyte interferon therapy in patients with cancer. *JAMA*, **252**, 938.

BISCAEGLIE, A.M., RUSTGI, V.K., HOOFNAGLE, J.H., DUSCHEIKO, G.M. & LOTZE, M.T. (1988). Hepatocellular carcinoma. *Ann. Intern. Med.*, **108**, 390.

CHLEBOWSKI, R.T., BRZECHWA-AJUDKIEWICZ, A., COWDEN, A., BLOCK, J.B., TONG, M. & CHAN, K.K. (1984). Doxorubicin (75 mg/m$^{-2}$) for hepatocellular carcinoma: clinical and pharmacokinetic results. *Cancer Treat. Rep.*, **68**, 487.

DUNK, A.A., IKEDA, T., PIGNATELLI, M. & THOMAS, H.C.C. (1986). Human lymphoblastoid interferon – *in vitro* and *in vivo* studies in hepatocellular carcinoma. *J. Hepatol.*, **2**, 419.

HENDERSON, I.G. & FREI, I., (1980). Adriamycin cardiotoxicity. *Am. Heart J.*, **99**, 671.

LAI, C.L. (1985). Chronic hepatitis B related diseases in Hong Kong. *Proc. Coll. Physicians Edinb.*, **15**, 157.

LAI, C.L., LAM. K.C., WONG, K.P., WU, P.C., & TODD, D. (1981). Clinical features of hepatocellular carcinoma: review of 211 patients in Hong Kong. *Cancer*, **47**, 2746.

LAI, C.L., WU, P.C., CHAN, G.C.B., LOK, A.S.F. & LIN, H.J. (1988). Doxorubicin versus no antitumour therapy in inoperable hepatocellular carcinoma: a prospective randomized trial. *Cancer*, **62**, 479.

LOK, A.S.F. & LAI, C.L. (1989). Alpha-fetoprotein monitoring in Chinese patients with chronic hepatitis B infection: role in the early detection of hepatocellular carcinoma. *Hepatology*, **9**, 110.

LONDON, W.T. (1981). Primary hepatocellular carcinoma – etiology, pathogenesis and prevention. *Human Pathol.*, **12**, 1085.

McDONALD, E.M., MANN, A.H. & THOMAS, H.C. (1987). Interferons as mediators of psychiatric morbidity: an investigation in a trial of recombinant $\alpha$-interferon in hepatitis-B carriers. *Lancet*, **ii**, 1175.

NAIR, P.V., TONG, M.J., KEMPF, R., CO, R., LEE, S.D. & VENTURI, C.L. (1985). Clinical serologic and immunologic effects of human leukocyte interferon in HBsAg-positive primary hepatocellular carcinoma. *Cancer*, **56**, 1018.

OLWENY, C.L.M., KATONGOLE-MBIDDE, E., BAHENDEKA, S., OTIM, D., MUGERWA, J. & KYALWAZI, S.K. (1980). Further experience in treating patients with hepatocellular carcinoma in Uganda. *Cancer*, **6**, 2712.

WHO (1983). *Prevention of Liver Cancer*. WHO Technical Report series. WHO: Geneva.

SACHS, E., DI BISCEGLI, A.M., DUSHEIKO, G.M. *et al.*, (1985). Treatment of hepatocellular carcinoma with recombinant leucocyte interferon: a pilot study. *Br. J. Cancer*, **52**, 105.

TANG, Z.-Y. (1985). General considerations on treatment of subclinical hepatocellular carcinoma. In *Subclinical Hepatocellular Carcinoma*, Tang Z.-Y. (ed) p.54 China Academic Publishers: Beijing.